# Two-stage Acetabular Impaction Bone Grafting: A Case Report

**DOI:** 10.5704/MOJ.2507.015

**Published:** 2025-07

**Authors:** V Selvaratnam, VJ Leong, S Gunainthran, S Chopra, AF Kassim

**Affiliations:** 1 National Orthopaedic Centre of Excellence for Research and Learning (NOCERAL), Department of Orthopaedic Surgery, Universiti Malaya, Kuala Lumpur, Malaysia; 2 Department of Orthopaedic Surgery, Hospital Sultanah Bahiyah, Alor Setar, Malaysia

**Keywords:** acetabular impaction bone grafting (AIBG), two-stage AIBG, irradiated femoral head allografts

## Abstract

Restoring bone loss in revision Total Hip Arthroplasty (THA) surgery is challenging. Acetabular impaction bone grafting (AIBG) allows the restoration of bone stock and normal hip biomechanics. AIBG is usually performed as a single-stage surgery with hip component implantation, as it is traditionally believed that adequate loading of the impacted graft is necessary for integration with the host bone thus preventing bone resorption. We present a case of a 73-year-old gentleman who presented with bilateral hip pain and reduced mobility. He was diagnosed with left THA aseptic loosening with acetabular protrusion and subsequently underwent a two-stage AIBG using irradiated femoral head allografts. The first stage was performed without acetabular loading, yet the allograft successfully integrated with the host bone. This is the first reported case of a two-stage AIBG demonstrating that acetabular loading may not be required for allograft integration to host bone. Therefore, AIBG may be performed as a two-stage procedure, when necessary, especially in the setting of complex revision hip surgeries and patients with poor bone stock.

## Introduction

Defects involving the acetabulum are classified as either contained or uncontained. The latter requires augmentation using acetabular augments or metal mesh to convert these to a contained stable cavity for acetabular impaction bone grafting (AIBG)^1^.

The successful use of AIBG in large defects relies on adequate loading of the impacted graft to remodel into living bone. Unloaded bone is thought to deteriorate and result in bone resorption. Therefore, AIBG is traditionally performed as a single-stage procedure with implantation of the hip components at the same sitting.

Initially, AIBG was used with a cemented socket. However, the current trend is to use this technique alongside modern uncemented shells which has shown good results^2^. However, some authors argue that cementing a polyethylene socket after AIBG, rather than the use of uncemented components, results in superior outcomes^3^. The use of an uncemented shell relies on contact with the host bone, and loading will occur preferentially at these points of contact. In contrast, a cemented socket also allows circumferential use of bone graft, in which the cement is in contact with the impacted graft over 100% of its interface. This allows restoration of bone stock in all areas of the acetabulum, something that is precluded by the necessity for host bone contact with cementless shells^4^.

We report a case of a patient who underwent a two-stage AIBG using irradiated femoral head allografts, resulting in successful bone graft integration at the second stage even though the AIBG was not loaded at the first stage. This is the first published report of a successful two-stage AIBG.

## Case Report

**Fig. 1:**
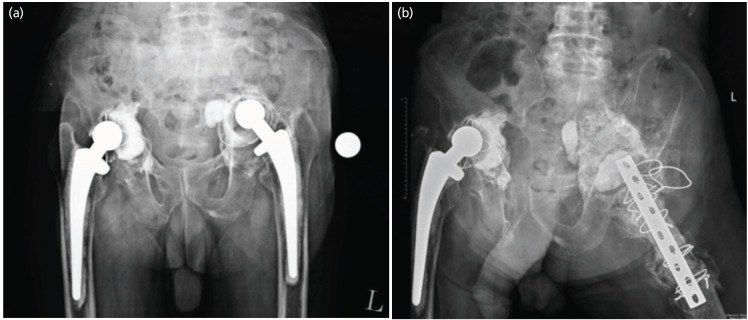
(a) AP Pelvic radiograph showing bilateral acetabular cup loosening with medial and superior protrusion of the acetabulum. (b) AP Pelvic radiograph showing AIBG integration.

This was a 73-year-old gentleman who had bilateral cemented total hip arthroplasty done in 1996. His hips were functioning well until August 2020 when he developed bilateral hip pain (left worse than right) with significantly reduced mobility. His hip range of movement was 25° to 70° on the left and 20° to 80° on the right. Radiographs showed bilateral acetabular cup loosening with a medial and superior protrusion of the acetabulum worse on the left (Fig. 1a). Inflammatory markers were normal and hip aspiration was clear.

We planned to reconstruct the acetabulum as a single-stage revision with AIBG and a cup cage construct. For the femoral side, either an in-cement revision or a restoration modular hip system was considered. A posterior approach was utilised. Intra-operatively (April 2021), the hip could not be dislocated due to the protrusion. Therefore, an extended trochanteric osteotomy to the tip of the stem was performed to remove the stem.

The femur was severely osteoporotic, resulting in a comminuted fracture of the femur requiring proximal femoral replacement (PFR) which was not available on standby. The cup and all the cement were removed on the acetabular side, revealing an intact medial membrane. A decision was made to perform AIBG against this membrane as part of a two-stage acetabular reconstruction.

A total of four irradiated femoral head allografts were used with bone chip sizes of around 8mm to 10mm obtained from femoral head allografts using rongeurs. Bone chips were applied in layers and each layer was well impacted with hemispherical impactors. A blob of bone cement mixed with 4g of Vancomycin powder was inserted in the acetabular cavity. The comminuted femur was held with a 4.5mm LCP plate with cables. The hip joint was then closed. The patient was non-weight bearing on his left lower limb.

**Fig. 2:**
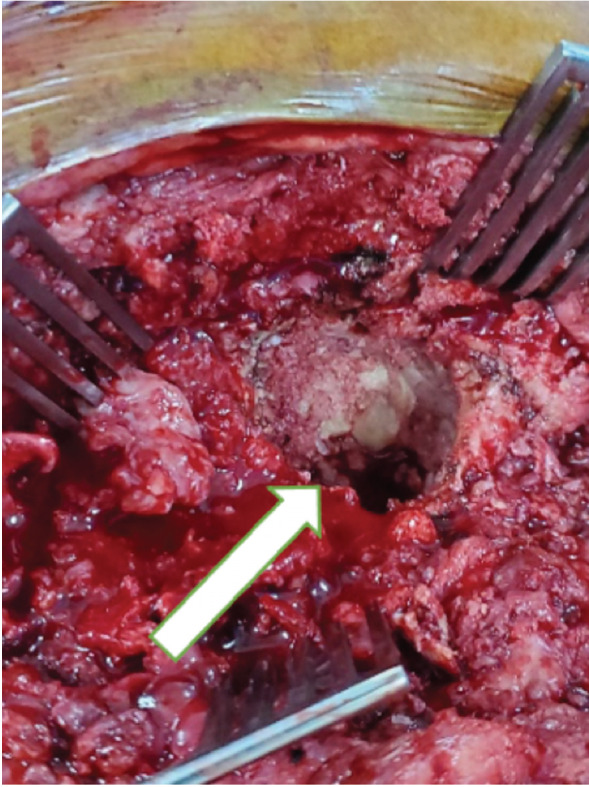
Intra-operative clinical photograph showing bone graft integration with a solid base.

**Fig. 3:**
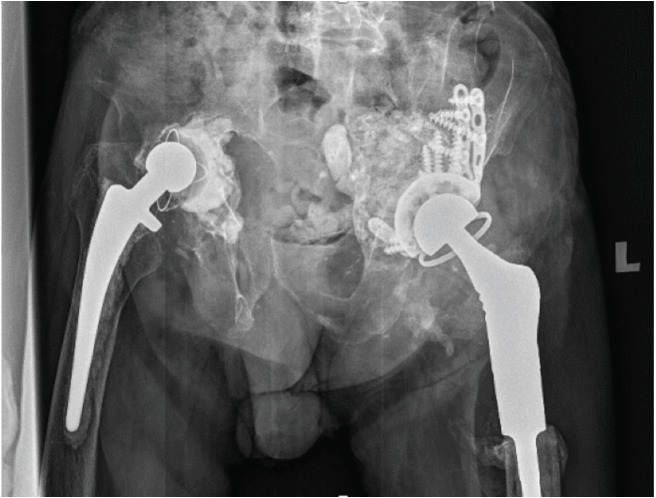
AP Pelvic radiograph showing a reconstructed left hip using a proximal femoral replacement and a cup cage with a cemented capture at two years follow-up.

A repeat radiograph was performed four months postoperatively which showed bone graft integration (Fig. 1b). AIBG integration was also confirmed by Computed Tomography (CT) scan. The second-stage surgery was performed in October 2021. Intra-operatively the bone graft integrated forming a solid base (Fig. 2). An uncemented trial cup was inserted but failed to get primary stability. Therefore, a cup cage was inserted, and a capture cup was cemented. The femur was reconstructed with a PFR (Fig. 3). There were no post-operative complications, and the patient was allowed full weight bearing as tolerated post-operatively with a walking frame. Ultimately, the patient was adherent to post-operative instructions and was able to tolerate a two-stage procedure. He was followed up at 2-weeks, 6-weeks, 3-months, 6-months, and yearly intervals thereafter.

## Discussion

The aim of an AIBG is to restore the structural integrity of the acetabulum and the anatomical hip centre. In our case, an acetabular defect secondary to acetabular protrusion warranted AIBG to reconstruct the acetabulum and improve the bone stock.

AIBG is usually performed as a single-stage procedure. This involves the preparation of bone grafts via morselized femoral head allografts followed by the impaction of these bone chips against the acetabulum or the underlying stiff membrane. Subsequently, a cup is inserted where the femoral component sits. Traditionally, this was a crucial step in determining the success of impaction grafting, as the femoral component acted as a transmission of force for the impaction of the acetabular bone grafts. This host bone contact was thought to be crucial in the integration of acetabular bone grafts, thus correcting the acetabular defect, improving the bone stock, and overall, creating a stable construct^3^.

However, as a PFR was not available to reconstruct the femur at the first stage, we decided to perform the acetabular reconstruction as a two-stage surgery. The first stage aimed to restore the cavitary defect with the impaction of morselized femoral head allografts against the underlying acetabular membrane.

Four months post-operatively, a repeat radiograph of the patient’s pelvis showed bone graft integration (Fig. 1b), and distinctly, the bone graft integrated with a solid base when observed intra-operatively (Fig. 2) at six months after the first stage. These findings suggest that the impacted acetabular bone grafts may not require loading to integrate with the host bone. Even in the absence of loading of the bone graft, integration was still possible, and the bone base was sufficiently stable for a second-stage surgery and full weight-bearing post-second-stage reconstruction.

Though it was unfortunate that a PFR was unavailable on standby, this case demonstrated that the loading of AIBG may not always be necessary for bone integration. Hence, where appropriate and necessary, an acetabular reconstruction with AIBG may be performed as a two-stage procedure.

A two-stage AIBG can therefore be utilised for severe acetabular defects with poor bone stock. The first stage optimises bone stock with bone grafting, and in the second stage, a reconstruction using either a cemented or an uncemented cup can be performed. These findings suggest that loading the AIBG at the first stage may not be necessary for bone integration.
